# Physical Activity, Bone Health, and Obesity in Peri-/Pre- and Postmenopausal Women: Results from the EPIC-Potsdam Study

**DOI:** 10.1007/s00223-015-0027-0

**Published:** 2015-06-25

**Authors:** Juliane Menzel, Romina di Giuseppe, Angelika Wientzek, Anja Kroke, Heiner Boeing, Cornelia Weikert

**Affiliations:** Research Group Cardiovascular Epidemiology, German Institute of Human Nutrition Potsdam-Rehbruecke, Nuthetal, Germany; Department of Epidemiology, German Institute of Human Nutrition Potsdam-Rehbruecke, Nuthetal, Germany; Department of Nutritional, Food and Consumer Sciences, Fulda University of Applied Sciences, Fulda, Germany; Institute for Social Medicine, Epidemiology and Health Economics, Charité University Medical Center, Berlin, Germany

**Keywords:** Bone mineral density, Physical activity, Broadband ultrasound attenuation, Bone loss, Obesity

## Abstract

Physical activity (PA) is suggested to increase the peak bone mass and to minimize age-related bone loss, and thereby to reduce the risk of osteoporosis. However, the relation between PA and bone health considering the obesity status is unclear so far. The present study examines the association between PA levels and calcaneal broadband ultrasound attenuation (BUA), particularly under consideration of obesity. Data from a population-based sample of 6776 German women from the EPIC-Potsdam cohort were analyzed. Calibrated PA data were used. Statistical analyses were stratified by menopausal and obesity status. Multiple linear regression was used to model the relationship between PA and BUA levels after adjustment for age, body mass index (BMI), smoking status, education, alcohol and calcium intake, and hormone use. Peri-/premenopausal had higher BUA levels (112.39 ± 10.05 dB/MHz) compared to postmenopausal women (106.44 ± 9.95 dB/MHz). In both groups, BUA levels were higher in the fourth compared to the lowest quartile of PA (*p* for trend < 0.05). In women with BMI < 30, but not BMI ≥ 30 kg/m^2^, PA remained positively associated with BUA levels (*p* for interaction = 0.03). However, when waist circumference higher than 88 cm or body fat percentage (BF %) measures above the median were used to define obesity, a significant positive relationship was also observed in women with BMI < 30 kg/m^2^ but with higher waist circumference or BF %. In conclusion, our results strengthen the hypothesis that PA has a positive influence on BUA levels, though dependent on weight.

**Electronic supplementary material:**

The online version of this article (doi:10.1007/s00223-015-0027-0) contains supplementary material, which is available to authorized users.

## Introduction

The prevalence of osteoporosis is increasing worldwide [1]. As a consequence, the incidence of osteoporotic fractures is projected to increase further along with the financial, social, and physical costs of the related treatments [1]. Therefore, a non-pharmacologic primary prevention is highly warranted [1]. Physical activity (PA) is one of the recommended strategies to maximize the peak bone mass in younger years and to minimize age-related bone loss, mostly effective via weight-bearing physical activities [1]. Nevertheless, minimizing falls and fractures risk through strength, flexibility, and balance training is also a central core of this demand [1]. In fact, PA modulates bone remodeling through mechanical stimuli, which results in improvements in mineralization and bone geometry [2].

Broadband ultrasound attenuation (BUA) is a non-invasive, bone health measurement technique at the os calcis without radiation. Previous studies have shown a positive association between PA and BUA levels [3–5]. Moreover, a number of studies have also confirmed that BUA is an independent predictor of fractures for both men and women particularly at low BUA values [3, 6, 7].

Besides higher PA levels, also higher body mass index (BMI) has been associated with higher BUA levels [8]. In particular, obesity has been suggested to protect against osteoporosis and several studies have shown a positive association between higher BMI and bone mineral density (BMD) [9–11]. However, inverse relationships between PA and obesity are also known [12]. Yet, how obesity influences the relation between PA and bone mineralization is, so far, unknown. Therefore, we aimed to investigate whether the association between PA and BUA differed according to obesity status.

In the present study, the associations between PA and BUA levels were investigated in a cross-sectional study using data from a large population-based sample of German women. All the analyses were conducted separately by peri-/premenopausal and postmenopausal status additionally considering the obesity status.

## Subjects and Methods

### Study Population

The European Prospective Investigation into Cancer and Nutrition (EPIC) Potsdam Study is part of the multicentre prospective cohort study conducted in 10 European countries, focusing on the relation between nutrition and various chronic diseases. Between 1994 and 1998, 27,548 participants (10,904 men and 16,644 women) aged 35–65 years were randomly selected from the general population of Potsdam and surrounding communities [13]. The recruitment process was based on residents´ registration offices, and the study participants were invited by mail to participate in the baseline examination [13]. At baseline, self-administered questionnaires on nutrition and lifestyle were filled out; computer-based interviews on lifestyle and medical history were conducted and physical examinations performed by trained personnel [13]. From 1996 until the end of the recruitment phase, quantitative ultrasound measurements (QUS) were part of the baseline examination in women. Therefore, from the total female population (*n* = 16,644), only 9711 women had QUS measurements. For the present study, we excluded 182 participants due to missing PA values, 117 due to age at baseline below 35 years, 2319 due to uncertain menopausal status, 302 due to surgical menopause, and 4 due to use of glucocorticoids or medication for the treatment of osteoporosis, respectively, both known to affect bone metabolism [14, 15]. Moreover, four peri-/premenopausal women with HRT intake and seven postmenopausal women taking oral contraceptives were excluded. The final study population consisted of 6776 women.

### Quantitative Ultrasound Measurement

The quantitative ultrasound measurements (QUS) were performed on the right os calcis using Achilles Plus Ultrasound Densitometer (Lunar Corporation, Madison, WI 53713, USA) by trained and quality-monitored personnel according to manufacturer instructions. Broadband ultrasound attenuation (BUA) was measured in decibel/megahertz (dB/MHz), defined as the slope of the signal attenuation versus the frequency curve in the usually measured range of 0.1–1 MHz [16]. In a substudy, 11 women were measured ten times within 3 weeks and a within person variation coefficient of 1.47 % of BUA was observed [17].

### Physical Activity Level

We used calibrated baseline EPIC-Germany PA data as described in Wientzek et al. [18, 19]. Briefly, the PA levels calculation was based on an Improved Physical Activity Index (IPAI) which was computed using information on weekly sports activities over the past 12 months in summer and winter, weekly cycling, weekly hours of watching television, as well as information on the type of occupational physical activity (sedentary, standing, manual, hard) [19]. Its performance was evaluated in a validation sample compared with the Cambridge Index and the Total PA Index. The IPAI is a valid physical activity index, showing higher correlations with accelerometer counts and physical activity energy expenditure than the Cambridge Index and the Total PA Index in EPIC Potsdam [19].

Next, a statistical model estimated from an EPIC subsample (*n* = 1339) was built based on a PA questionnaire and objectively measured PA data (acceleration counts). Segmented regression coefficients adjusted for sex, age, and BMI were fitted to baseline PA data in EPIC-Germany [18].

### Anthropometry

Trained and quality-monitored personnel took the anthropometric measurements (weight, height, and waist circumference) with participants wearing only light underwear and no shoes. Body weight was measured with electronic digital scales, accurate to 100 g (Soehnle, type 7720/23, Murrhardt, Germany) and the height to the nearest 0.1 cm by using a flexible anthropometer. BMI was calculated as body weight (kg) divided by squared height (m^2^). Obesity was defined according to BMI measures based on the World Health Organization cutoff points as follows: BMI (BMI < 30 kg/m^2^; BMI ≥ 30 kg/m^2^). The skinfold thickness at biceps, triceps, subscapular, and suprailiac on the right body site was measured with a standard caliper (Lange, Cambridge, MD, USA). Based on these skinfold thicknesses, body density was derived from the regression equations of Durnin and Womersly, which were used to calculate the percentage of body fat according to Siri’s formula [17].

### Assessment of Dietary Intake, Lifestyle Characteristic, and Other Covariates

Dietary intake was assessed with a self-administered food frequency questionnaire including questions on frequency and portion size of 148 items consumed during the previous year [20]. Information on educational level, smoking habits, and medical and reproductive history was obtained by PC-assisted face-to-face interviews. Menopausal status was assessed according to self-reported information about menstrual status and history, e.g., natural menopause or surgical menopause. Postmenopausal status is defined as follows: women with <3 months menstruation in the last year, without hysterectomy and/or ovariectomy, and only HRT use with 12 consecutive months without menstruation. Women, who have experienced ≥3 months with menstruation in the last year, without hysterectomy and/or ovariectomy, and no use of HRT, were defined as peri-/premenopausal women. Self-reported medication over the past 4 weeks prior to study enrolment was used to identify users of oral contraceptive, hormone replacement therapy (HRT), glucocorticoids, and specific drugs against osteoporosis.

### Statistical Analysis

All analyses were performed separately for peri-/premenopausal (*n* = 4230) and postmenopausal (*n* = 2546) women. Normally distributed variables (BUA, age, BMI) were reported as mean and standard derivation. Right skewed variables (intake of calcium and alcohol) were reported as median and interquartile range, and log transformed for the analyses. Categorical variables were reported as percentage (smoking status, educational level, oral contraceptive, and HRT use). Multivariable linear regression models were used to estimate the relationships between BUA and PA levels, with BUA as continuous dependent variable. Based on a review of the literature, the following variables were identified as potential confounders: age (continuous), BMI (continuous), smoking status (non-smoker, ex-smoker, current smoker), educational level (unskilled or skilled, technical college, university degree), log-transformed alcohol and log-transformed calcium intake, use of oral contraceptive (yes/no), and HRT (yes/no), respectively, for peri-/premenopausal and postmenopausal women. For a better understanding of the relationship between PA and BUA, menopausal status-specific quartiles of PA were also calculated. Comparisons between each quartile using the lowest (Q1) as reference were tested using the Dunnett`s method. Multiplicative interactions between PA and menopausal status as well as PA and BMI (continuous) were tested in the fully adjusted model by including the cross product terms. To explore the data for nonlinear relationships, polynomial terms were included in the regression models. With the aim to evaluate the clinical relevance of the relationship between PA and BUA levels, a different regression model was built. In particular, the beta coefficients of the mean values of all covariates were fixed, and BUA values were predicted for different values of PA. For postmenopausal women, the mean PA value observed in the highest quartile of PA in the premenopausal group was used. The same sensitivity analysis was performed according to obesity status.

Since in the present study, obesity was defined as BMI ≥ 30 kg/m^2^, and considering that the effectiveness of BMI to discriminate fat from lean mass is inadequate [21], we performed additional analyses using body fat percentage (BF %) and waist circumference measures to define obesity (normal: waist ≤88 cm; raised: waist >88 cm). However, as so far no threshold of BF % for defining obese and non-obese has been set [22], these groups were determined by baseline BF % above and below the median (35.5 %).

To further investigate whether the associations between BUA and PA levels were modified according to both BMI and BF % and in order to evaluate whether the use of BMI to classify obesity leads to misclassification, analyses were additionally stratified by BMI (BMI < 30 kg/m^2^; BMI ≥ 30 kg/m^2^) and BF % (above or below the median) or waist circumference (≤88 cm or >88 cm) using the models outlined above. Formal tests for interaction were performed between BMI, BF %, and PA levels by including a three-level interaction: BMI × PA levels, BF % × PA levels, and BMI × BF % and BMI × BF % × PA levels interaction terms in the fully adjusted model. The same tests were performed using waist circumference instead of BF %.

All statistical analyses were performed using SAS software, version 9.4 (SAS institute, Cary, N.C., USA).

## Results

The general characteristics of the 6776 women, stratified by menopausal status, are shown in Table 1. Peri-/premenopausal women were younger, and had higher BUA and PA levels. No differences were observed between oral contraceptive user (*n* = 2753) and non-user (*n* = 1477) (data not shown). The interaction term between PA and menopausal status was statistically significant (*p* = 0.02); therefore, all analyses were stratified by peri-/pre- and postmenopausal status.

**Table 1:** Characteristics of the study population according to menopausal status (EPIC-Potsdam study, women, *n* = 6776)

	Peri-/premenopausal women (*n* = 4230)	Postmenopausal women (*n* = 2546)	*p*
BUA (dB/MHz)	112.39 ± 10.05	106.44 ± 9.95	<0.0001
Age (years)	40.78 ± 4.37	58.85 ± 4.12	<0.0001
BMI (kg/m^2^)	24.45 ± 4.30	27.26 ± 4.64	<0.0001
Physical activity (counts/min/day)	41.10 ± 4.81	31.68 ± 5.12	<0.0001
Waist (cm)	76.96 ± 10.67	84.94 ± 11.37	<0.0001
Smoking status (%)			<0.0001
Non-smoker	2873 (67.9)	2104 (82.6)	
Ex-Smoker	354 (8.4)	145 (5.7)	
Current smoker	1003 (23.7)	297 (11.7)	
Educational level (%)			<0.0001
Unskilled or skilled	1519 (35.9)	1275 (50.1)	
Technical College	1126 (26.6)	812 (31.9)	
University degree	1585 (37.5)	459 (18.0)	
Oral contraceptive intake (%)	1477 (34.9)	–	<0.0001
Hormone replacement therapy (%)	–	568 (22.3)	<0.0001
Nutrients intake			
Calcium intake (g/day)	0.74 (0.59, 0.95)	0.71 (0.56, 0.92)	<0.0001
Alcohol consumption (g/day)	5.89 (2.23, 11.56)	3.93 (1.14, 9.01)	<0.0001
Protein intake (g/day)	65.4 (54.6, 78.9)	64.5 (54.4, 77.2)	0.0412

Variables are expressed as percentage, or mean and standard deviation, or median and interquartile range

PA was linearly related to BUA. The positive association observed between PA and BUA remained after adjustment for age, BMI, smoking status, educational attainment, log-transformed alcohol and calcium intake, and hormone use in both groups. In particular, each unit increase in the PA level was significantly associated with 0.38 and 0.30 dB/MHz higher BUA levels, respectively, in peri-/premenopausal and postmenopausal women (*p* < 0.0001). Postmenopausal women had lower PA levels compared to peri-/premenopausal women. Analyses performed according to menopausal status-specific PA quartiles showed the same linear relationship between PA and BUA levels in peri-/premenopausal and postmenopausal women (Table 2). In both groups, the mean BUA levels in the second, third, and fourth quartiles were significantly different from the mean of the referent quartile (Q1). In peri-/premenopausal women, the mean BUA level in the lowest quartile was 110.67 dB/MHz 95 % CI (109.80, 111.54), and in the fourth quartile, the mean BUA was 113.89 dB/MHz 95 % CI (113.08, 114.69) (*p* values Dunnett’s test < 0.009). Postmenopausal women had values of 109.08 dB/MHz 95 % CI (109.96, 110.19) in the top PA level quartile versus values of 105.46 dB/MHz 95 % CI (104.31, 106.60) in the bottom PA quartile (*p* values Dunnett’s test < 0.007). An additional analysis in postmenopausal women showed that increasing the PA level from 31.7 to 46.8 counts/min/day could increase the BUA values from 106.4 to 111.0 dB/MHz. As shown in Table 3, obesity status modifies the relationship between PA and BUA values (*p* for interaction = 0.03). Stratified analyses by obesity status (BMI < 30 vs. BMI ≥ 30 kg/m^2^) showed that peri-/premenopausal obese women had higher BUA (115.44 ± 9.71 dB/MHz) levels compared to non-obese (112.04 ± 10.03 dB/MHz). Also postmenopausal women with BMI ≥ 30 kg/m^2^ had higher BUA levels (108.45 ± 10.14 dB/MHz) than non-obese ones (105.79 ± 9.80 dB/MHz). However, the positive association between PA and BUA only remained in both non-obese peri-/premenopausal and postmenopausal women (Table 3). A sensitivity analysis in obese women (BMI ≥ 30) showed that only increasing PA to levels much higher than those observed in the highest PA quartile of non-obese women (BMI < 30) would lead to clinical meaningful increase in the BUA levels in both groups (data not shown). Stratified sensitivity analyses by obesity status using four BMI categories (BMI < 25.0 kg/m^2^, 25.0 kg/m^2^ < BMI < 30.0 kg/m^2^, 30.0 kg/m^2^ < BMI < 35.0 kg/m^2^, BMI ≥ 35.0 kg/m^2^) showed positive associations between PA and BUA levels only in non-obese (BMI < 25.0 kg/m^2^, 25.0 kg/m^2^ < BMI < 30.0 kg/m^2^) peri-/premenopausal and postmenopausal women. However, the number of very obese women was rather small which may have limited the power to detect associations (Supplementary Tables 1s and 2s).

**Table 2:** Quartiles of physical activity with adjusted BUA values according to menopausal status

	*n*	PA (counts/min/day)	BUA (dB/MHz)	*p* linear trend
Peri-/premenopausal women (*n* = 4230)^a^				
Q1	1057	35.67 (33.40, 37.19)	110.67 CI (109.80,111.54)	<0.0001
Q2	1058	39.84 (39.11, 40.65)	112.13 CI (111.46, 112.80)*	
Q3	1058	42.95 (42.27, 43.67)	113.41 CI (112.71, 114.11)*	
Q4	1057	46.35 (45.30, 47.93)	113.89 CI (113.08, 114.69)*	

Postmenopausal women (*n* = 2546)^b^				
Q1	636	25.90 (23.70, 27.33)	105.46 CI (104.31, 106.60)	<0.0001
Q2	637	30.16 (29.41, 30.98)	107.27 CI (106.30, 108.23)*	
Q3	637	33.44 (32.63, 34.38)	107.94 CI (106.97, 108.91)*	
Q4	636	37.60 (36.20, 39.25)	109.08 CI (109.96, 110.19)*	

Variables are expressed as adjusted mean and 95 % confidence interval, or median and interquartile range. Adjustment: age, BMI, smoking status, education, alcohol intake log transformed, calcium intake log transformed, oral contraceptive use^a^, HRT^b^

* Significantly different compared to Q1 (ANOVA with Dunnett adjustment)

**Table 3:** Quartiles of physical activity with adjusted BUA values stratified by menopausal status and BMI categories (BMI < 30, BMI ≥ 30)

	*n*	PA (counts/min/day)	BUA (dB/MHz)^a^	*p* linear trend
Peri-/premenopausal women (*n* = 3795) BMI < 30^a^				
Q1	948	37.37 (35.72, 38.40)	110.04 CI (109.17, 110.91)	<0.0001
Q2	949	40.63 (39.88, 41.40)	111.43CI (110.71, 112.14)*	
Q3	949	43.39 (42.73, 44.04)	113.22 CI (112.49, 113.95)*	
Q4	949	46.61 (45.60, 48.15)	113.70CI (112.86, 114.53)*	

Peri-/premenopausal women (*n* = 435) BMI ≥ 30^a^				
Q1	108	28.73 (26.18, 30.03)	116.72 CI (114.16, 119.29)	0.97
Q2	109	32.33 (31.67, 33.11)	115.75 CI (113.67, 117.82)	
Q3	109	34.85 (34.28, 35.65)	114.98 CI (112.83, 117.13)	
Q4	109	38.24 (37.36, 39.50)	117.05 CI (114.77, 119.33)	

Postmenopausal women (*n* = 1918) BMI < 30^b^				
Q1	479	28.64 (27.33, 29.70)	104.78 CI (103.60, 105.96)	<0.0001
Q2	480	31.84 (31.17, 32.57)	105.97 CI (104.90, 107.04)	
Q3	480	34.67 (33.90, 35.16)	107.23 CI (106.17, 108.29)*	
Q4	479	38.42 (37.14, 39.92)	108.45 CI (107.22, 109.68)*	

Postmenopausal women (*n* = 628) BMI ≥ 30^b^				
Q1	157	21.57 (19.70, 22.93)	109.79 CI (107.30, 112.27)	0.24
Q2	157	25.38 (23.74, 16.16)	108.98 CI (106.82, 111.16)	
Q3	157	28.05 (27.23, 28.68)	108.79 CI (106.59, 110.99)	
Q4	157	31.56 (30.26, 32.95)	111.77 CI (109.44, 114.11)	

Variables are expressed as adjusted mean and 95 % confidence interval, or median and interquartile range. Adjustment: age, BMI, smoking status, education, alcohol intake log transformed, calcium intake log transformed, oral contraceptive use^a^, HRT^b^

* Significantly different compared to Q1 (ANOVA with Dunnett adjustment)

A three-level interaction analysis by including a BMI × BF % × PA, or BMI × waist circumference × PA, interaction term in the fully adjusted model showed a significant interaction (*p* < 0.0001). Stratified analyses by obesity/BF % status showed positive significant relationships only among non-obese women with BF % above or below the median, while no significant associations were found in obese women with BF % either above or below the median (Table 4). Similar results were observed when waist circumference categories were used instead of BF % (Table 4).

**Table 4:** Multiple linear regression model on the association between PA and BUA stratified by body fat percentage/waist, BMI, and menopausal status

	Peri-/premenopausal women						Postmenopausal women					
	BMI < 30			BMI ≥ 30			BMI < 30			BMI ≥ 30		
	*n*	β-coefficient^a^	*p*	*n*	β-coefficient^a^	*p*	*n*	β-coefficient^b^	*p*	*n*	β-coefficient^b^	*p*
BF % < Median	2389	0.43195	<0.0001	4*	–	–	949	0.32951	0.005	15	0.71665	0.7
BF % > Median	1405	0.36795	0.0005	420	0.12314	0.5	969	0.34944	0.002	606	0.19205	0.2
Waist ≤ 88 cm	3605	0.40446	<0.0001	66	−0.20801	0.7	1580	0.29201	0.001	54	0.04410	0.9
Waist > 88 cm	190	0.59369	0.03	369	0.12581	0.5	338	0.54542	0.002	574	0.18652	0.2

BF % missing *n* = 19; BF % median = 35.5 %; Variables are expressed as mean and standard deviation; Adjustment: age, BMI, smoking status, education, alcohol intake log transformed, calcium intake log transformed, oral contraceptive use^a^, HRT^b^

* Calculation not possible due to a low number of individuals within these strata

## Discussion

Calcaneal broadband ultrasound attenuation was studied in relation to PA levels, taking into consideration the obesity status, in peri-/premenopausal and postmenopausal women. In this cross-sectional analysis, we observed a significant positive association between PA levels and BUA in peri-/pre- and postmenopausal women. To date, only a few studies investigated the relationship between PA and BUA levels in women applying complex PA instruments [23, 24]. In particular, in the present study, we used a complex PA score including comprehensive information on weekly sports, cycling, hours of television watched, and different types of occupational PA. This latter point is particularly important considering that other PA domains, such as activity in the workplace, remain very often unexplored [25]. PA instruments that only focus on leisure time PA may indeed underestimate the true activity level. In fact, for many people, a significant proportion of their lifetime exposure to intensive PA will occur in the workplace [26].

Our findings are generally consistent with observations made by other epidemiological studies among women of different ethnicities, using complex PA assessment tools as well. These studies mainly observed a positive association between PA and BUA levels [4, 23, 24, 27], only Brahm et al. and Brunner et al. noticed no association [28, 29].

In line with other population-based studies, mean BUA values observed in the present study reached 112 dB/MHz in premenopausal [30, 31] and 106 dB/MHz in postmenopausal women [3, 4, 29]. Interestingly, mean BUA levels of 107.2 dB/MHz were observed in a group of women that developed a fracture versus BUA levels of 111.2 dB/MHz in women who did not [32]. In particular, in this latter prospective study of 422 mainly postmenopausal women, the authors observed a 43 % increased risk of fracture per 1 SD decrease in the BUA levels after a mean follow-up of 2.6 years [32]. The postmenopausal women had lower PA level compared to the peri-/premenopausal women. But interestingly, based on our data, we calculated that in postmenopausal women, an increase of mean PA levels from 31.7 to 46.8 counts/min/day, i.e., the mean value observed in the fourth PA quartile of premenopausal women, would induce a change in BUA values from 106.4 dB/MHz (values at risk for future fractures) to 111.0 dB/MHz (values observed in the non-fracture group of Huopio et al. study). This estimation suggests that increasing the levels of PA might positively influence BUA levels and lower the risk of fractures.

Obesity has been discussed as a protective factor for osteoporotic fractures [33]. However, a meta-analysis based on 25 prospective cohorts, with data of 368, 610 women with over 2.2 million person-years of follow-up, observed that high BMI is a risk factor for both humerus and elbow fracture [33]. Moreover, it has been discussed that obese subjects were more likely to fracture their ankle [33]. Though the reason for these site‐specific associations is unknown, it has been suggested that it may be related to an increased risk of falling or a greater load upon bones in falls [33].

To our knowledge, this is the first epidemiological study investigating the influence of obesity on the relation between PA and BUA levels. To classify the obesity status we used BMI, the most common anthropometric measure to diagnose obesity, waist circumference, and BF % measures.

However, to date, the majority of studies have primarily shown associations between BMI only, as a measure of obesity, and BMD. Indeed, a higher BMI has been positively associated with BMD, assessed with dual energy X-ray absorptiometry (DEXA) or BUA [10, 11, 24, 31, 34]. We also observed that obese women had higher BUA levels than non-obese ones. Interestingly, some studies observed both a positive association between weight/BMI and bone mineral density and a negative relationship between fat mass and bone mineral density within given weight/BMI categories, measured with DEXA [10, 11, 35, 36]. These results indicate two diverging aspects of obesity and bone health. On the one hand, the positive weight-bearing loading related to obesity is a strong mechanical stimulus for improvement of bone mineralization and bone geometry. On the other hand, obesity carries a series of detrimental aspects associated with fat mass [10]. In particular, visceral fat has been associated with increased levels of proinflammatory cytokines, which in turn promote bone resorption and osteoporosis [37]. Therefore, we were particularly interested in investigating the relation between PA and BUA levels taking obesity status into account. Surprisingly, no significant associations were observed between PA and BUA levels in obese peri-/pre- and postmenopausal women, using BMI. To investigate whether these findings were the result of a misclassification bias, likely occurring when BMI is used as a measure of obesity, additional analyses were performed using both BF % and established waist circumference cutoff points to further define obesity. However, we detected no significant associations between PA and BUA levels among obese women (BMI ≥ 30 kg/m^2^) with BF % either above or below the median, or waist circumference either above or below the cutoff points. Indeed these results could suggest that weight bearing on the bones, mainly, independently from body fat, lean body mass, muscles or other components, might be related to higher BUA levels. Moreover, the PA levels on average were lower in obese women compared to non-obese. However, we estimated that obese women should increase their PA to very high levels, much higher than those observed in the highest PA quartile of non-obese in both groups, in order to reach a clinical meaningful increase in the BUA levels. Nevertheless, even though in correctly classified obese women based on BMI and waist circumference, or BF % measurements, we did not observe a significant association between PA and BUA levels, presumably because of habitual loading and possible circulating estrogens [38], PA may still remain a recommendation to maintain bone health. Indeed, low PA levels and high BMI or waist circumference have consistently been shown to be predictors of several comorbidities, including cardiovascular disease [39, 40]. Therefore, women should always be encouraged to stay physically active and adhere to a healthy BMI/waist circumference for long-term health.

This study has some limitations that need to be addressed. First, BUA was derived from QUS measurement on the right os calcis and we did not include measures at other sites or used additional techniques. Despite DEXA is the most frequently used technique for assessing bone mineral density, BUA has been validated several times against this method. [41] Interestingly, in the study of Taal and colleagues, BUA measures performed at the os calcis were highly correlated with femoral neck and total hip DEXA measures [42]. Furthermore, Moayyeri et al. observed a similar performance between BUA and DEXA measures in prediction of long-term fractures risk in the elderly [43]. Overall, these findings suggest that BUA can be used as a valid, inexpensive, easy, and quick alternative assessment tool for bone health, and most importantly, without radiation [1, 44]. The os calcis is a weight-bearing bone [24]. During weight-bearing exercises, when the minimum effective strain at the bone site is exceeded, the strain or deformation at this area becomes an osteogenic stimulus [45]. Based on this, the physical stimulus leads to a shift in trabecular orientation and density of the bone [46]. The force exerted by the ground on a body in contact with it is known as ground reaction force (GRF). GRF with every heel-strike is maximal at the os calcis, so heel might be an appropriate site for evaluation of the effects of PA on bone [24]. Second, the used PA index does not include non-sports activities of daily living. Finally, the current study is limited to Caucasian women, and thus, the results cannot be generalized to men or to other racial/ethnic groups. Finally, this is a cross-sectional study not allowing causal inferences.

Strengths of our study include the population-based sample, the high number of participants, thus ensuring a suitable comparison between peri-/premenopausal and postmenopausal women, and the availability of high quality data as a result of the standardized procedures.

In conclusion, this study shows a positive association between PA and BUA levels in both peri-/premenopausal and postmenopausal women, albeit dependent on obesity status. Though a direct relationship between PA and BUA levels was observed only in non-obese, indeed these findings suggest that women should stay active to increase bone mass in younger years and to maintain bone mass in the elderly.

## Electronic supplementary material

Supplementary material 1 (DOC 45 kb)

Supplementary material 2 (DOC 45 kb)
