# Supplementary material for: Physical Activity, Bone Health, and Obesity in Peri-/Pre- and Postmenopausal Women: Results from the EPIC-Potsdam Study
Source: Calcif Tissue Int. 2015 Jun 25;97(4):376–84. doi: 10.1007/s00223-015-0027-0 (PMC4564447; doi:10.1007/s00223-015-0027-0)
Supplement: Supplementary file 1 — Supplementary material 1 (DOC 45 kb) [file 223_2015_27_MOESM1_ESM.doc]

Table 1s: Quartiles of physical activity with adjusted BUA values stratified by BMI categories (BMI < 25, 25 < BMI < 30, 30 < BMI < 35, BMI ≥ 35) in peri-/premenopausal women

| **Peri-/premenopausal women (n=2732) BMI < 25.0** | | | | |
| --- | --- | --- | --- | --- |
|  | n | PA [counts/min/day] | BUA [dB/MHz] | P linear trend |
| Q1 | 683 | 39.21 (38.08, 40.02) | 109.90 CI (108.76, 110.65) | <0.0001 |
| Q2 | 683 | 42.18 (41.51, 42.76) | 110.44 CI (109.60, 111.28) |  |
| Q3 | 683 | 44.38 (43.82, 44.97) | 112.30 CI (111.61, 113.32)* |  |
| Q4 | 683 | 47.35 (46.38, 48.72) | 112.97 CI (112.03, 113.91)* |  |
|  |  |  |  |  |
| **Peri-/premenopausal women (n=1063) 25 < BMI< 30** | | | | |
|  | n | PA [counts/min/day] | BUA [dB/MHz] | P linear trend |
| Q1 | 265 | 34.91 (33.61, 35.63) | 112.22 CI (110.74, 113.70) | 0.0009 |
| Q2 | 266 | 37.63 (37.06, 38.14) | 113.28 CI (111.93, 114.63) |  |
| Q3 | 266 | 38.57 (39.12, 40.15) | 113.98 CI (112.62, 115.34)* |  |
| Q4 | 266 | 42.54 (41.80, 43.93) | 115.75 CI (114.29, 117.20)* |  |
|  | | | | |
| **Peri-/premenopausal women (n=320) 30 < BMI < 35** | | | | |
|  | n | PA [counts/min/day] | BUA [dB/MHz] | P linear trend |
| Q1 | 80 | 30.63 (29.78, 31.44) | 116.75 CI (113.97, 119.53) | 0.78 |
| Q2 | 80 | 33.58 (33.07, 34.10) | 115.39 CI (112.93, 117.85) |  |
| Q3 | 80 | 35.87 (35.40, 36.51) | 114.59 CI (112.14, 117.04) |  |
| Q4 | 80 | 38.68 (37.98, 39.90) | 117.60 CI (115.05, 120.16) |  |
|  |  |  |  |  |
| **Peri-/premenopausal women (n=115) BMI ≥ 35.0** | | | | |
|  | n | PA [counts/min/day] | BUA [dB/MHz] | P linear trend |
| Q1 | 28 | 23.11 (20.78, 23.39) | 114.98 CI (109.54, 120.42) | 0.63 |
| Q2 | 29 | 28.65 (27.88, 29.22) | 117.88 CI (113.70, 122.05) |  |
| Q3 | 29 | 31.51 (30.71, 32.15) | 116.99 CI (112.59, 121.38) |  |
| Q4 | 29 | 34.09 (33.55, 35.59) | 113.31 CI (108.69, 117.93) |  |
| Variables are expressed as adjusted mean and 95%-confidence interval, or median and interquartile range. Adjustment: age, BMI, smoking status, education, alcohol intake log transformed, calcium intake log transformed, oral contraceptive use  *Significantly different compared to Q1 (ANOVA with Dunnett adjustment) | | | | |
