# Supplementary material for: Physical Activity, Bone Health, and Obesity in Peri-/Pre- and Postmenopausal Women: Results from the EPIC-Potsdam Study
Source: Calcif Tissue Int. 2015 Jun 25;97(4):376–84. doi: 10.1007/s00223-015-0027-0 (PMC4564447; doi:10.1007/s00223-015-0027-0)
Supplement: Supplementary file 2 — Supplementary material 2 (DOC 45 kb) [file 223_2015_27_MOESM2_ESM.doc]

Table 2s: Quartiles of physical activity with adjusted BUA values stratified by BMI categories (BMI < 25, BMI 25 < 30, BMI 30 < 35, BMI ≥ 35) in postmenopausal women

| **Postmenopausal women (n=905) BMI < 25.0** | | | | |
| --- | --- | --- | --- | --- |
|  | n | PA [counts/min/day] | BUA [dB/MHz] | P linear trend |
| Q1 | 226 | 31.22 (29.93, 31.20) | 103.50 CI (101.83, 105.17) | 0.0004 |
| Q2 | 226 | 34.27 (33.61, 34.71) | 106.34 CI (104.74, 107.93)* |  |
| Q3 | 227 | 36.57 (35.85, 37.37) | 107.24 CI (105.66, 108.83)* |  |
| Q4 | 226 | 39.79 (38.90, 40.98) | 107.54 CI (105.86, 109.23)* |  |
|  |  |  |  |  |
| **Postmenopausal women (n=1013) 25 < BMI< 30** | | | | |
|  | n | PA [counts/min/day] | BUA [dB/MHz] | P linear trend |
| Q1 | 253 | 27.46 (26.73, 28.31) | 105.26 CI (103.71, 106.80) | 0.002 |
| Q2 | 253 | 30.16 (29.69, 30.74) | 107.16 CI (105.96, 108.62) |  |
| Q3 | 254 | 32.38 (31.86, 33.08) | 106.04 CI (104.63, 107.45) |  |
| Q4 | 253 | 35.63 (34.81, 37.03) | 108.93 CI (107.38, 110.47)* |  |
|  | | | | |
| **Postmenopausal women (n=467) 30 < BMI < 35** | | | | |
|  | N | PA [counts/min/day] | BUA [dB/MHz] | P linear trend |
| Q1 | 116 | 24.02 (22.62, 24.81) | 109.79 CI (107.30, 112.29) | 0.11 |
| Q2 | 117 | 26.71 (26.19, 27.11) | 107.90 CI (105.52, 110.29) |  |
| Q3 | 117 | 28.86 (28.40, 29.46) | 110.26 CI (107.85, 112.68) |  |
| Q4 | 117 | 32.13 (30.93, 33.65) | 111.62 CI (109.05, 114.20) |  |
|  |  |  |  |  |
| **Postmenopausal women (n=161) BMI ≥ 35.0** | | | | |
|  | n | PA [counts/min/day] | BUA [dB/MHz] | P linear trend |
| Q1 | 40 | 18.35 (16.97, 19.42) | 110.61 CI (104.47, 116.75) | 0.16 |
| Q2 | 40 | 21.46 (20.79, 21.99) | 109.99 CI (104.10, 115.88) |  |
| Q3 | 41 | 23.70 (23.19, 24.49) | 107.04 CI (101.38, 112.70) |  |
| Q4 | 40 | 26.88 (26.05, 28.23) | 106.67 CI (100.81, 112.53) |  |
| Variables are expressed as adjusted mean and 95%-confidence interval, or median and interquartile range. Adjustment: age, BMI, smoking status, education, alcohol intake log transformed, calcium intake log transformed, oral contraceptive use  *Significantly different compared to Q1 (ANOVA with Dunnett adjustment) | | | | |
